# EBV Reactivation in Transplant Recipients following SARS-CoV-2 Infection: A Retrospective Study

**DOI:** 10.3390/pathogens12121435

**Published:** 2023-12-10

**Authors:** Lucia Federica Stefanelli, Marianna Alessi, Caterina Di Bella, Maria Elena Billo, Ludovica Viola, Maddalena Gnappi, Elisabetta Bettin, Martina Cacciapuoti, Lorenzo A. Calò

**Affiliations:** 1 Nephrology, Dialysis and Transplantation Unit, Department of Medicine, Padova University Hospital, 35128 Padova, Italymarianna.alessi@aopd.veneto.it (M.A.); mariaelena.billo@aopd.veneto.it (M.E.B.); ludovica.viola@aopd.veneto.it (L.V.); maddalena.gnappi@aopd.veneto.it (M.G.); elisabetta.bettin@aopd.veneto.it (E.B.); martina.cacciapuoti@aopd.veneto.it (M.C.); 2 Kidney and Pancreas Transplantation Unit, Department of Surgery, Oncology and Gastroenterology, Padova University Hospital, 35128 Padova, Italy; caterina.dibella@aopd.veneto.it

**Keywords:** Epstein Barr virus, kidney transplant, SARS-CoV-2, COVID-19

## Abstract

Reactivation and primary infection with a high Epstein Barr Virus (EBV) DNA level in kidney transplant patients could cause severe complications, including the development of Post-Transplantation Lymphoproliferative Disease (PTLD). While in the general population the reactivation of EBV after SARS-CoV-2 infection has been reported, very few data are available in transplant recipients. Our retrospective study aimed to evaluate a possible EBV reactivation in kidney transplant patients following SARS-CoV-2 infection and a possible impairment of the immune system. In addition, the effects of changes in immunosuppressive therapy on EBV DNA reactivation and vaccination were also evaluated. A total of 166 kidney transplant patients followed at the Kidney–Pancreas Transplant Ambulatory Nephrology Unit at Padova University Hospital were retrospectively considered for an observation period of 6 months from January 2020 to April 2023. EBV DNA level was measured by Rt-PCR and evaluated 6 months before and after SARS-CoV-2 infection. Patients’ serological states were established via quantification of anti-VCA and anti-EBNA (chemiluminescence). Patients’ immune systems were characterized by CD4^+^/CD8^+^ lymphocyte ratio (flow cytometry). EBV DNA was reactivated in 50% of the 166 patients with COVID-19 who completed the study. Older patients with more severe forms of COVID-19 had higher EBV reactivation (*p* < 0.05). EBV reactivation significantly increased in patients with severe SARS-CoV-2 infection requiring hospitalization compared to patients managed at home (*p* < 0.001). CD4^+^/CD8^+^ lymphocyte ratio was reduced in patients with a younger age of transplant (*p* < 0.01) and on a higher dose of steroids (*p* < 0.01). The results of our study confirm the role of immunodepression, especially in recent transplant patients and those on high steroids, in EBV reactivation. These results combined with the few available in the literature might contribute to providing an optimal management of immunosuppressive treatment for these patients in order to obtain an immune state unfavorable to the activation of latent viruses, including EBV.

## 1. Introduction

Kidney transplantation represents the replacement treatment of choice in End Stage Kidney Disease (ESKD). Renal survival in the first year after transplantation is, in fact, greater than 95% in living donor transplants and 89% in deceased donor transplants [1].

Significant advances in surgical technique and induction and maintenance immunosuppression regimens have improved allograft outcomes. Nonetheless, infections remain a leading cause of complications after kidney transplantation [2]. A number of factors affect the onset of the infections, including specific donor and recipient factors, such as a preexisting infection or immunity, the use of antimicrobial prophylaxis, and the net state of immunosuppression [3].

Among the wide spectrum of potential pathogens that affects immunocompromised hosts, there are viruses, in particular Epstein Barr Virus (EBV). EBV is a ubiquitous viral pathogen, with a seroprevalence of more than 90% in adults.

In transplant recipients, both acute infection and reactivation of latent infection may lead to clinical pictures associated with non-neoplastic viral replication or to EBV mediated neoplastic transformation, including Post-Transplantation Lymphoproliferative Disease (PTLD) [4]. Infected patients develop a clinical picture of infectious mononucleosis, in particular amongst younger patients, sometime associated with a life-threatening sepsis-like presentation [5]. EBV-PTLD represents the most serious complication in kidney-transplanted patients with an incidence of 1–5% [6]. Diagnosis of EBV is performed by quantitative monitoring of EBV by Rt-PCR [7]. A high EBV DNA level has also been associated with a higher risk of PTLD in patients at high immunological risk [8], and the velocity of EBV PCR rise, may have a better positive predictive value for EBV PTLD, with an increasing titer suggestive of disease [9]. Taking into account national and international guidelines, PTLD treatment is based on the type and severity of the underlying disease and needs a multidisciplinary approach [10,11]. 

Severe acute SARS-CoV-2 has rapidly spread around the world since the beginning of the COVID-19 pandemic, fueled by the emergence of several variants. A feature of COVID-19 that differs from other respiratory infections could be its multi-system involvement with complications and long-term sequelae (Long-COVID) [12]. Long-COVID refers to a group of conditions and symptoms involving different systems, such as hematological, cardiological, gastrointestinal and nervous systems associated with general constitutional symptoms such as asthenia, fever and fatigue [13]. While a number of studies have suggested the role of autoimmune factors and persistence of viral fragments in the development of Long-COVID, the role of other latent host viruses such as EBV has not been considered or ruled out. 

Although recent studies have hypothesized the possible interaction between SARS-CoV-2 and EBV, the mechanism of this interaction still remains unknown [14]. Kidney transplant recipients have a high risk of death from COVID-19, and due to their comorbidities and immunocompromised state it is reasonable to expect more complications, such as infections or the reactivation of latent viruses such as EBV [15]. However, most data come from studies on the general population. The findings of these studies, in fact, do not establish any definitive conclusion, while the mechanistic role of COVID-19 in the reactivation of latent EBV, in particular in kidney transplant recipients, is of clear importance due to the negative role played by this infection both on graft and patient survival [4]. 

Our retrospective monocentric study adds to the few studies available in the literature in order to provide further information regarding the reactivation or primary infection by EBV following COVID-19 infection both in hospitalized and non-hospitalized kidney transplant recipients. In addition, a possible alteration of the immune system and/or reactivation of EBV in immunosuppressed patients has also been considered via the evaluation of the relationship between CD4^+^ and CD8^+^ lymphocyte subpopulations. Finally, the effect of vaccination and changes in immunosuppressive therapy on the reactivation of EBV DNA have also been evaluated.

## 2. Materials and Methods

### 2.1. Patients

In this retrospective monocentric study, 166 single, double or combined kidney–pancreas or kidney–liver transplant recipients followed at the Kidney–Pancreas Transplant Ambulatory Unit of Padova University Hospital have been considered.

**Figure 1:**
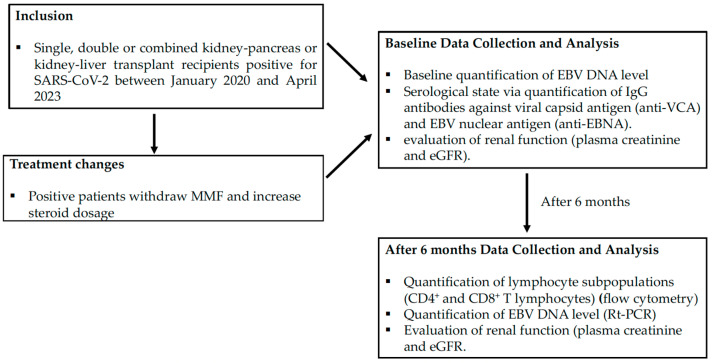
Design and flow of the study.

The patients were considered between January 2020 and April 2023. All patients were positive for SARS-CoV-2 and their data were extracted from medical records. The patients were followed for six months. Exclusion criteria included: age < 18 years; preexisting diagnosis of chronic EBV infection; and previous positive diagnosis of PTLD EBV. Figure 1 summarizes the design and the flow of the study.

### 2.2. Methods

The laboratory data included the evaluation of renal function with plasma creatinine, (μmol/L) and eGFR (mL/min) and the characterization of the lymphocyte subpopulations (CD4^+^ and CD8^+^ T lymphocytes) using flow cytometry (Aquios CL System, Beckman Coulter, Indianapolis, IN, USA). The serological state of the patients was analyzed with the quantification of IgG antibodies against the viral capsid antigen (anti-VCA) and against the EBV nuclear antigen (anti-EBNA) via chemiluminescence immunoassays. 

Nasopharyngeal swabs samples were performed in all hospitalized and ambulatory patients and were tested using Rt-PCR to identify SARS-CoV-2 infection. 

EBV DNA level was determined using Rt-PCR and a value equal to or higher than 1000 copies/mL was considered as a positive. The EBV DNA was calculated at two different times, before SARS-CoV-2 infection and after 6 months from the infection. All the collected samples were processed at the Central Laboratory of the Padova University Hospital.

Ethical review and approval were waived for this study as required for a retrospective clinical investigation. Patients were not exposed to any risk by the irreversible anonymization of data. The anonymization process prevented any possible transmission of sensible data, protecting subjects’ privacy.

### 2.3. Statistical Analysis

Statistical analysis was performed using the IBM SPSS Statistics 22 software (IBM, Armonk, NY, USA). Data are presented as absolute values and their percentages vs. total number. The Shapiro–Wilk test and the Kolmogorov–Smirnov test were used to check the normal distribution. Differences between proportions were analyzed by Pearson’s Chi-squared test of independence, while the Kruskal–Wallis test was used for continuous variables. The Mann–Whitney U test was used for the analysis of quantitative variables. Values of 5% or less (*p* < 0.05) were considered significant.

## 3. Results

### 3.1. Patient Cohort

In this study we considered a total of 166 single, double or combined kidney–pancreas or kidney–liver transplant recipients followed at the Kidney–Pancreas Transplant Ambulatory Unit of Padova University Hospital with a median age of 56 years (23–88). The median transplant vintage was 5 years (1–32). The median creatinine at the time of EBV DNA reactivation was 126 μmoL/L and eGFR, calculated according to the eGFR CKD-EPI formula, was 57.7 mL/min (15–130).

The number of SARS-CoV-2 positive swabs were 25 in 2020 (14.45%), 41 in 2021 (23.70%), 109 in 2022 (63%) and 7 (4.04%) in 2023 (up to April 2023).

Data analysis revealed that 162 patients (97.6%) were on maintenance therapy with a calcineurin inhibitor (CNI), 137 patients (82.5%) were using mycophenolic acid and 22 patients (12.86%) were receiving mTOR Inhibitors (mTORi). Out of 166 patients, 142 (85.5%) were on a low steroid dose. 

In patients infected with SARS-CoV-2, CNI therapy was discontinued in 19 patients (11.4%). Mycophenolic acid and mTORi were discontinued in 93 (56%) and 6 patients (3.6%), respectively. Steroid dosage was increased in 103 patients (62%).

In the 166 patients included in this study, 130 received the vaccine for SARS-CoV-2 (78.3%); hospitalization for severe respiratory failure occurred in 29 cases (17.5%), while the other 137 patients presented only minor symptoms not requiring hospitalization. The most represented comorbidity was hypertension (116 hypertensive patients, 69.9%), while 26 patients (15.7%) had diabetes mellitus and 21 patients (12.7%) had a known oncological history. These latter had noninvasive skin cancer after transplantation, which was not active at the time of the study. Table 1 summarizes the demographic and clinical characteristics of the 166 transplant patients included in the study.

### 3.2. EBV DNA Detection and Outcomes

**Figure 2:**
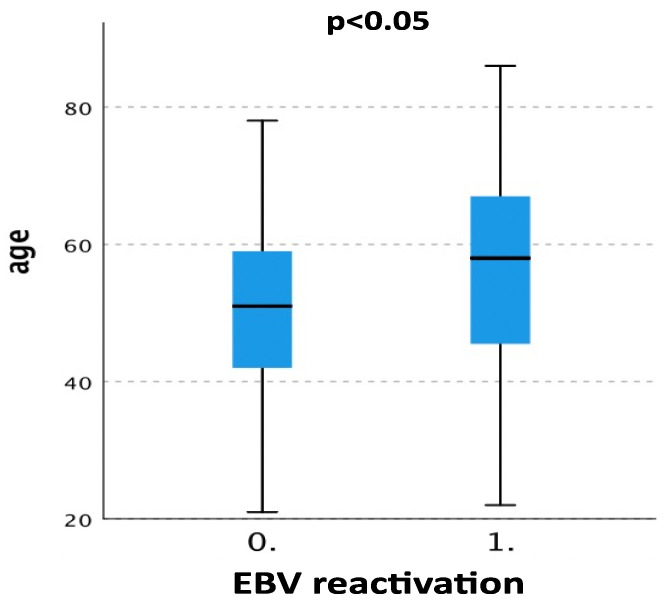
Box plot showing the association between higher age and increased EBV DNA reactivation. (0 = no reactivation; 1 = reactivation).

EBV DNA was reactivated in 83 patients (50%), and reactivation was higher in older patients with a median age > 60 years compared with patients with a median age < 60 years (41.3% vs. 21.3%; *p* < 0.05) (Figure 2, Table 2), while the age of the transplant and creatinine did not influence the number of reactivations.

In terms of viral reactivation, modifications in immunosuppressive therapy and vaccination did not show statistically significant differences, while EBV reactivation was significantly increased in patients with severe SARS-CoV-2 infection requiring hospitalization compared to patients managed at home: 16 patients out of 30 requiring hospitalization had viral reactivation vs. only 31 patients out of 137 managed at home (53.3% vs. 22.6%; *p* < 0.001, Table 2).

### 3.3. Lymphocyte Subpopulation Count and Viral Serology

**Figure 3:**
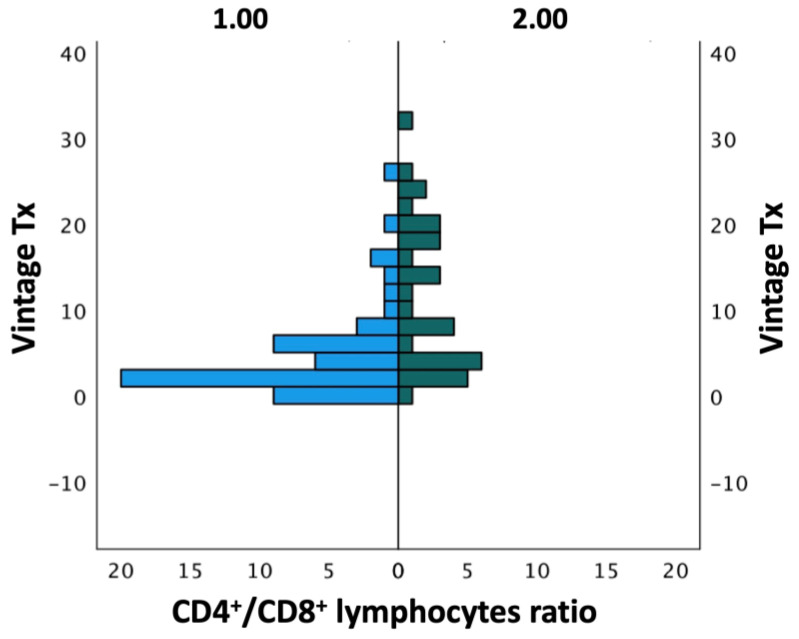
Mann–-Whitney U test showing greater immunosuppression (CD4^+^/CD8^+^ lymphocyte ratio ≤1) in transplant patients with younger transplant age (1 = CD4^+^/CD8^+^ lymphocyte ratio in patients with younger transplant age (less than 1 year); 2 = CD4^+^/CD8^+^ lymphocyte ratio in patients with older transplant age).

The assessment of the net immunosuppression state was performed via the evaluation of the CD4^+^/CD8^+^ T lymphocyte ratio, considering a patient with a ratio ≤ 1 as immunosuppressed. Patients with a more recent transplant age were found to be more immunocompromised compared to patients with an older transplant age: 45 patients out of 65 vs. 7 patients out of 23 (72% vs. 30.4%; *p* < 0.01) (Figure 3).

Age and creatinine did not influence the CD4^+^/CD8^+^ lymphocyte ratio. On the contrary, modifications in immunosuppressive therapy significantly influenced the CD4^+^/CD8^+^ lymphocyte ratio. In particular, patients whose steroid dose had been increased had a reduced CD4^+^/CD8^+^ lymphocyte ratio (79% of the patients vs. 53%; *p* < 0.01, Table 2). The presence of comorbidities and hospitalization did not influence the immunological state.

## 4. Discussion

This study reports the demographic and clinical characteristics of kidney transplant patients affected by COVID-19, and evaluates and considers the possible determinants in the reactivation of EBV DNA in this type of patient during a 6 month observation period after SARS-CoV-2 infection.

EBV is a common viral infection in transplant recipients and could have negative consequences on both patients’ life and graft survival; moreover, the B lymphocytes, a target of the virus, could undergo an uncontrolled proliferation resulting in a polymorphic or monomorphic EBV+ PTLD [16]. In transplant patients, immunosuppressive therapy and inflammatory stress causing immunosuppression via the alteration of the CD8^+^ T lymphocyte and macrophage function may lead to complications, in particular, an increased EBV-related neoplastic risk [17]. While the relationship between immunosuppression and the reactivation of latent viruses in transplant recipients is well known [4], less is known in these patients regarding the impact of COVID-19 on EBV reactivation and immunological state. 

Several studies have hypothesized a possible interaction between SARS-CoV-2 and EBV in the general population. A possible mechanism could involve the decrease in CD8^+^ lymphocyte cells, which are the primary cells responsible for immunity against EBV infection [18]. In addition, EBV may influence the occurrence of Long-COVID via the synergistic disruption of cellular and mitochondrial pathways [19]. To our knowledge, no study has focused on a possible EBV reactivation and immune system change post-COVID-19 in kidney transplant patients. In 2021 Chen et al. documented, for the first time, EBV reactivation in COVID-19 patients in the general population [20], while only Basic-Jukic et al. mentioned EBV reactivation in 27% of transplant patients [21]. In the 6 month period of our retrospective study, 50% of transplant patients had EBV DNA reactivation. This is quite relevant coming from the considerable total number of 166 transplant recipients. In addition, our study is the first which has considered both patients hospitalized for severe SARS-CoV-2 infection and home-managed patients and has also evaluated the effects of vaccination. 

The analysis of the data obtained in our patients both hospitalized and managed at home and regardless of the presence or absence of comorbidities, has also shown that EBV reactivation following COVID-19 was higher in older transplant patients compared to younger patients. In addition, although Basic-Jukic et al. showed that hypertension and diabetes were risk factors not only for infection but also for viral reactivation [21], in the patients of our cohort with the same comorbidities, EBV DNA rate was not different compared with patients with no risk factors. 

Our study compared, for the first time, EBV viral reactivation in transplant recipients vaccinated with the SARS-CoV-2 mRNA vaccine and in those not vaccinated. However, no difference was found regarding the rate of EBV reactivation between vaccinated and unvaccinated transplant patients, while a reactivation of EBV DNA after two doses of the SARS-CoV-2 mRNA vaccine was shown in 4.1% of transplant patients by Musialik et al. [22]. 

The relationship between immunosuppressive therapy, in particular maintenance therapy with CNI and steroids and induction therapy with antithymoglobulins (ATG), and the reactivation of latent viruses is well known [2]. In our study, therapeutic modifications, in particular the increase of steroids or the withdrawal of antimetabolites or CNIs, were not associated with the reactivation of EBV DNA. Studies conducted in the general population such as the study of Paolucci et al., showed more significant EBV reactivation in Intensive Care Unit (ICU) patients [23] and Lenher et al. showed that 78% of COVID-19 patients with respiratory insufficiency requiring intensive ventilation had greater EBV reactivation [24]. These data agree with those of our study, in which hospitalized patients showed a significant difference in terms of viral reactivation compared to patients managed at home, although there was no interaction between illness severity in hospitalized patients and the receipt of higher steroid doses, while, as reported below, a higher level of immunosuppression in terms of a greater reduction of the CD4^+^/CD8^+^ lymphocyte ratio was observed in patients under a high steroid dose, which was not related to EBV reactivation. 

Our study has also investigated in transplant patients the possible influence of the reduction of T lymphocytes, NK cells and the CD4^+^CD8^+^ lymphocyte ratio, associated with COVID-19, on the greater tendency for EBV DNA reactivation in order to explore a possible influence of the altered immune system on EBV reactivation. Our study shows a significantly greater reduction in the CD4^+^/CD8^+^ lymphocyte ratio in patients with a more recent transplant who have a higher level of immunosuppression, including a higher dose of steroids, regardless of age, renal function, comorbidities and vaccination. In addition, a greater reduction in the CD4^+^/CD8^+^ lymphocyte ratio, and therefore a greater immunosuppression, was observed in patients under a high steroid dose and, although higher doses of steroids in our patients was apparently not related to the observed EBV reactivation, data in the literature show that high doses of steroids are associated with greater immunosuppression and a tendency to reactivate latent viruses [25].

Limitations of our study are as follows: (1) the reduction of T lymphocytes, NK cells and the CD4^+^/CD8^+^ lymphocyte ratio and the greater EBV activation have been found in our patients in terms of an association, while the demonstration of a cause–effect type of relationship or the identification of a specific correlation could give more strength to the results; (2) there is a lack of information regarding possible differences in the activation of both SARS-CoV-2 and EBV during the different waves of the pandemic in relation to vaccine doses; (3) there is a lack of a clear demonstration of the importance of higher doses of steroids for EBV reactivation.

The strength of our study is the number of patients in our cohort compared to the few studies on EBV reactivation in transplant patients with COVID-19 available in the literature. In addition, our study is the first that has analyzed, in transplant patients with COVID-19, the relationship between variations of the immune state and the possible activation of latent viruses.

## 5. Conclusions

A reactivation of Herpes Viruses and, in particular, of EBV after a clinically severe form of SARS-CoV-2 infection has been reported in the general population. Our study has investigated in transplant patients a reactivation of latent and pro-oncogenic viruses such as EBV as a consequence of COVID-19.

Our results showed, in a considerable number of transplant patients, a reactivation of EBV DNA after COVID-19 infection associated with a reduction of CD4^+^/CD8^+^ lymphocyte ratio in those with higher immunosuppression. This could contribute to providing transplant patients with an optimal management of immunosuppressive therapy in order to obtain an immune state unfavorable to the activation of latent viruses including EBV.

## Figures and Tables

**Table 1:** Demographic and clinical characteristics of the 166 transplant patients included in the study.

Total Number of Patients	166
**Demographic**	
- Male	104 (62.6%)
- Female	62 (37.4%)
- Median age	56 y (23–88)
**Comorbidity**	
- Hypertension	116 (69%)
- Diabetes	26 (15.7%)
- Oncologic History	21 (12.7%)
- Hospitalization	29 (17.5%)
**Type of treatment**	
- CNI	162 (7.6%)
- MMF (Acid Mycophenolic)	137 (82.5%)
- Corticosteroid	142 (85.5%)
**Withdrawal of treatment**	
- CNI	19 (11.4%)
- MMF	93 (56%)
- mTORi	6 (11.4%)
**Increase of treatment**	
- Steroid	103 (62%)
**Patients positive for COVID-19**	
- COVID-19 positive patients in 2020	25 (14.45%)
- COVID-19 positive patients in 2021	41 (23.70%)
- COVID-19 positive patients in 2022	100 (60.2%)
**Patients with vaccination for SARS-CoV-2**	
- Vaccine	130 (78.3%)

**Table 2:** EBV reactivation rate in older vs. younger patients (>60 y.o. vs. <60 y.o.) and CD4^+^/CD8^+^ ratio in the patients with more recent vs. older transplant vintage.

		Statistical Significance
	Patient median age	
	>60 y vs. <60 y	*p* < 0.05
**EBV Reactivation**		
	Hospitalization for severe COVID-19 vs. home-managed	
		*p* < 0.001
	More recent transplant vintage vs. older transplant vintage	
		*p* < 0.01
**CD4^+^/CD8^+^ Ratio**		
	Steroid dose increased vs. unchanged	
		*p* < 0.01

## Data Availability

Although subjects included in the study were not exposed to any risk by the irreversible anonymization of data, to make sure subjects’ privacy is protected from any possible transmission of sensible data, data are stored by the corresponding author.
